# Design of a microscopic electrical impedance tomography system for 3D continuous non-destructive monitoring of tissue culture

**DOI:** 10.1186/1475-925X-13-142

**Published:** 2014-10-06

**Authors:** Eun Jung Lee, Hun Wi, Alistair Lee McEwan, Adnan Farooq, Harsh Sohal, Eung Je Woo, Jin Keun Seo, Tong In Oh

**Affiliations:** Department of Computational Science and Engineering, Yonsei University, 120-749 Seoul, South Korea; Department of Biomedical Engineering and Impedance Imaging Research Center, Kyung Hee University, 46-701 Yongin, Korea; The School of Electrical and Information Engineering, University of Sydney, NSW2006 Sydney, Australia

**Keywords:** Non-destructive monitoring, Label-free, Electrical impedance tomography, Three-dimensional impedance image, Tissue culture monitoring, Projected image reconstruction algorithm

## Abstract

**Background:**

Non-destructive continuous monitoring of regenerative tissue is required throughout the entire period of *in vitro* tissue culture. Microscopic electrical impedance tomography (micro-EIT) has the potential to monitor the physiological state of tissues by forming three-dimensional images of impedance changes in a non-destructive and label-free manner. We developed a new micro-EIT system and report on simulation and experimental results of its macroscopic model.

**Methods:**

We propose a new micro-EIT system design using a cuboid sample container with separate current-driving and voltage sensing electrodes. The top is open for sample manipulations. We used nine gold-coated solid electrodes on each of two opposing sides of the container to produce multiple linearly independent internal current density distributions. The 360 voltage sensing electrodes were placed on the other sides and base to measure induced voltages. Instead of using an inverse solver with the least squares method, we used a projected image reconstruction algorithm based on a logarithm formulation to produce projected images. We intended to improve the quality and spatial resolution of the images by increasing the number of voltage measurements subject to a few injected current patterns. We evaluated the performance of the micro-EIT system with a macroscopic physical phantom.

**Results:**

The signal-to-noise ratio of the developed micro-EIT system was 66 dB. Crosstalk was in the range of -110.8 to -90.04 dB. Three-dimensional images with consistent quality were reconstructed from physical phantom data over the entire domain. From numerical and experimental results, we estimate that at least 20 × 40 electrodes with 120 *μm* spacing are required to monitor the complex shape of ingrowth neotissue inside a scaffold with 300 *μm* pore.

**Conclusion:**

The experimental results showed that the new micro-EIT system with a reduced set of injection current patterns and a large number of voltage sensing electrodes can be potentially used for tissue culture monitoring. Numerical simulations demonstrated that the spatial resolution could be improved to the scale required for tissue culture monitoring. Future challenges include manufacturing a bioreactor-compatible container with a dense array of electrodes and a larger number of measurement channels that are sensitive to the reduced voltage gradients expected at a smaller scale.

## Background

Due to the rapid developments in materials science and biotechnology, the regeneration of tissues for replacing failing or malfunctioning organs has dramatically advanced [1]. New therapeutic approaches using the functions of embryonic, fetal, and adult stem cell progenitors in tissue engineering and regenerative medicine are promising strategies to treat Parkinson and Alzheimer diseases, muscular degenerative disorders, chronic liver and heart failures, as well as diverse aggressive cancer types [2, 3]. These tissues are commonly cultured *in vitro* using their high self-renewal capacity and potential to generate differentiated cell progenitors, before transplantation to the host *in vivo*. It is important to create an artificial environment enabling cells to induce tissue regeneration during the culture period [4]. Considering the shortage of donors and the high costs,a continuous non-destructive monitoring of *in vitro* tissue culture is required for improved feedback controls to increase productivity and quality of the final implant.

The natural healing of cartilage rarely occurs after damage because cells in lacunae are surrounded by cartilaginous matrix molecules and cannot migrate to a wound site [5]. Autologous chondrocytes implantation (ACI) has been used for the regeneration of articular cartilage [6]. This *in vitro* cell culture approach involves isolation of autologous chondrocytes from cartilage of a non-load bearing site and *in vitro* expansion before injection to the site of damaged cartilage [7–9]. Engineered cartilage has been also investigated through *in vitro* tissue culture. Since the articular cartilage consists of four specific zones which have different properties due to the matrix morphology and biochemical compositions after growth [10], we can estimate the quality, functionality, and physiological state of regenerating tissues using these characteristics. We may control growth factors and other bioactive agents to achieve their respective constructs and functionality [11]. All of these cell and tissue culture techniques require a non-destructive monitoring and imaging method to assess the physiological property of tissues and their distribution in real-time. Noting that the conductivity of chondrocytes changes dramatically during the culture period and the conductivities in the four zones are different due to morphological differences and compositions [10], we may devise a non-destructive monitoring method based on electrical impedance imaging techniques.

Electrical impedance tomography (EIT) can visualize the internal impedance distribution of a conducting domain such as a biological object, a tissue sample or the human body. Noninvasive weak currents are injected into the domain and the resulting boundary current-voltage data sets are measured to determine the transfer impedance values from which the internal impedance distribution is reconstructed as a cross-sectional image [12]. EIT has the potential to electrically monitor cell growth, proliferation, differentiation, migration and apoptosis based on the microscopic impedance distribution. We may also apply the EIT technique to *in vitro* tissue culture for its monitoring. It is a non-destructive, non-invasive, label-free, multi-dimensional (space, time and frequency), and direct imaging method.

A well known limitation of EIT is the relatively low spatial resolution of the reconstructed impedance images due to the ill-posed nature of the image reconstruction problem using surface measurements. There have been several suggestions to improve the spatial resolution for its use at the microscopic scale. Microscopic impedance imaging methods using conventional EIT techniques have been developed to investigate the growth and migration of cells during cultivation [13–16]. In order to enhance the resolution in the reconstructed images, they used a large number of miniaturized electrodes with the same structure of the conventional EIT system or with micro-scanning for space division multiplexing [16–21]. All electrodes were used for current generation and voltage measurement to create a large number of combinations. However, this required a complicated circuit to support both current injection and voltage sensing for each electrode. This led to degraded performance of the measurement system because each electrode was loaded with a multiplexer and the contact impedance of current-driving electrode was high due to its small surface area. This approach was hampered by technical difficulties of using a limited number of electrodes and an increased impact of the nonlinearity which was the variation of voltage difference due to the internal impedance changes [12].

Recently, a microscopic impedance imaging system called the KHU (Kyung Hee University) Mark1 micro-EIT system was introduced [22–24]. It used a unique electrode configuration and data collection method that separated the driving and sensing electrodes. Even though it used a projected image reconstruction algorithm with better resolution than the conventional microscopic impedance imaging methods, there were some problems such as underestimation of the volume and artifacts around the secondary current driving electrodes. This might be due to the positions of highly conductive voltage sensing electrodes and secondary current-driving electrodes in the hexahedral sample container.

In this study, we aim to investigate a new design of current-driving electrodes and an improved image reconstruction algorithm. We will first explain the new design concept and details of the pilot system called the KHU Mark2 micro-EIT system. We will then present promising experimental results using a macro phantom setup. Based on the experimental and also simulation results, we will estimate the required number of electrodes for monitoring *in vitro* tissue culture for engineered articular cartilage.

## Methods

### Electrode configuration in a sample container

**Figure 1:**
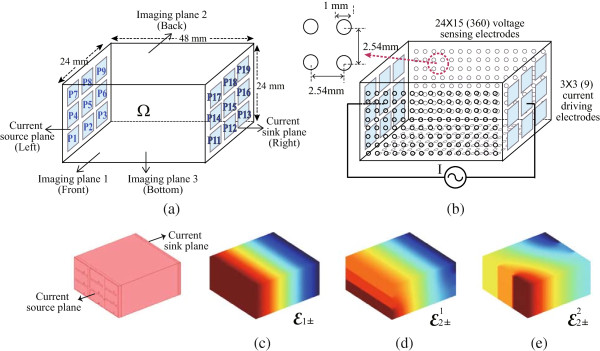
**Design of the sample container included voltage sensing electrodes and current driving electrodes for the Kyung Hee University (KHU) Mark2 micro-EIT system.** The EIT system injects current on surface electrodes and senses voltages on an additional set of surface electrodes. **(a)** The dimensions of a sample container and each 3 × 3 current driving electrodes on the current source plane and the current sink plane. **(b)** 24 × 15 voltage sensing electrodes on three imaging planes and dimensions for each sensing electrode. **(c)** Simulation of the voltage distributions subject to the primary current  and **(d and e)** simulation results for two secondary currents, , .

In order to obtain the most information of internal impedance from surface measurements, we installed arrays of small voltage sensing electrodes and large current driving electrodes on five sides of the sample container. The top was left open for tissue manipulations. Larger electrodes are required for current driving to reduce the contact impedance and improve current density uniformity. Figure 1(a) and (b) show the sample container and the location of the current driving electrodes and voltage sensing electrodes. Let *P*_*k*_, *k* = 1,⋯,9 and *k* = 11,⋯,19, be the large square current driving electrodes placed on both shorter ends of the container in Figure 1(a). We refer to the longer container sides with voltage sensing electrodes as the imaging planes 1, 2, and 3. These are respectively, the front, back, and bottom sides of the container.

The selection of *m* × *n*-array for injecting current was carefully chosen to control the flow of current. The current electrodes can be combined to increase their surface area thereby reducing the overall contact impedance of the compound electrode. We installed 9 gold-coated solid square electrodes, in a 3 × 3-array, for injecting current on the current source plane and current sink plane. 8 rows of 15 voltage sensing electrodes were installed on each imaging plane. This leads to a total of 360 (24 × 15) voltage sensing electrodes as shown in Figure 1(b). In this configuration currents flow approximately along the tangential direction of the voltage sensing electrodes. To produce a projected impedance image on each imaging plane, we require at least two data sets obtained when injecting at least two linearly independent currents. The two currents are referred to as the primary () and secondary currents (). We carefully choose paired combinations of the nine current source and nine sink electrodes. We chose  and  to inject the primary current so that we can create a uniform parallel current density distribution inside the container when it is filled with homogeneous conducting medium (Figure 1(c)). For the secondary current injection, we chose the solid metal plates on both ends so that the dominant direction of the secondary current is linearly independent to the primary current. For instance, we chose ,  to produce the secondary current for the projected images on the imaging plane 1 and 2 (Figure 1(d)). For imaging plane 3, we inject the secondary current between  and  to maximize the detectability of impedance changes (Figure 1(e)). In both cases, we used the same primary measurement data when applying currents through .

This electrode configuration and current injection method combined with multiple voltage measurements are unique in the KHU Mark2 micro-EIT system. They help to enhance the spatial resolution of the reconstructed images and detectability for anomalies inside the container.

### Projected image reconstruction algorithm

In order to improve the spatial resolution of impedance images, we applied the projected image reconstruction algorithm introduced in [24]. Let *Ω* be the hexahedral cuboid sample container as shown in Figure 1. We set  to be a pair of driving electrodes for the primary current and  to be a pair of driving electrodes for the secondary current injection as described in the previous section. This configuration provides a large number of voltage measurements from the sensing electrode array subject to the two current injections. The major advantages related with the separation of current driving and voltage sensing electrodes are the simplification of hardware complexity and the improvement of measurement accuracy from a large number of small voltage sensing electrodes. This is advantageous even though it limits the number of current patterns. In this work we only use two current patterns that result in only two sets of Dirichlet data for the projected image reconstruction, yet the spatial resolution is improved by the large number of voltage sensing electrodes.

We modified the projected image reconstruction algorithm to fit our new model. This included using the logarithm of admittivity distribution instead of the inverse of the admittivity distribution. When injecting the low-frequency current of amplitude *I* through two pairs of current driving electrodes () for the primary (*j* = 1) and the secondary (*j* = 2) cases, the resulting potential *u*^*j*^ satisfies the following equations:
1

where *γ* is the admittivity distribution and **n** is outward unit normal vector on the boundary of *Ω*, *∂Ω*. From the first equation in (1), a simple calculation yields
2

Since the voltage data is only available on the sensing surfaces, we may assume *∂*_*z*_*γ* ≈ 0 and  on the sensing surfaces. Then, the equation (2) can be rewritten in a matrix-vector formulation as follows
3

in which *γ*^∗^ is the projected value of admittivity distribution in the sample container to the sensing surface and . Now we invert the matrix *J* (this is why we need to induce two linearly independent currents) and take a divergence in *xy*-coordinate, ∇_xy_·, on both sides of the equation in (3) to obtain
4

After obtaining the three, two dimensional projected images of the interior admittivity distribution, we apply the standard back-projection scheme to create a three dimensional image of the admittivity distribution in the sample container.

### The Kyung Hee University (KHU) Mark2 micro-EIT system

**Figure 2:**
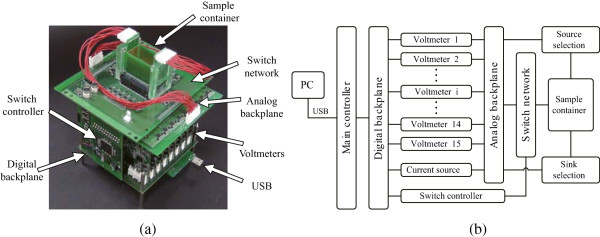
**Design of the KHU Mark2 micro-EIT system.** **(a)** Implemented KHU Mark2 micro-EIT system, and **(b)** its functional block diagram. The system connects to a PC over USB. The digital backplane controls the current source, each measurement channel and organizes data acquisition. Each channel contains a voltmeter that is connected to the analog backplane. These are connected to the sample container through a switch network and associated controller.

We developed the KHU Mark2 micro-EIT system to confirm the new electrode configuration, algorithm and measurement method as depicted in Figure 2. The system was descended from the KHU Mark1 micro-EIT system in terms of technical details, such as digital waveform generation, Howland current source with multiple generalized impedance converters and digital phase-sensitive demodulators [23]. However, it required a significant modification to obtain the projected images from the new container with the new electrode configuration and measurement method. This included modifying the switch network to allow any combination of current driving electrodes. The switch network also required modification to efficiently measure the 360 differential voltage values for each current injection using 15 voltmeters. The current source module included the automatic self-calibration circuit to maximize its output impedance using the droop method proposed by Cook *et al.* [25]. Within the calibration steps, we removed the DC offset in the current output and compensated the phase delay at each operating frequency [26].

**Figure 3:**
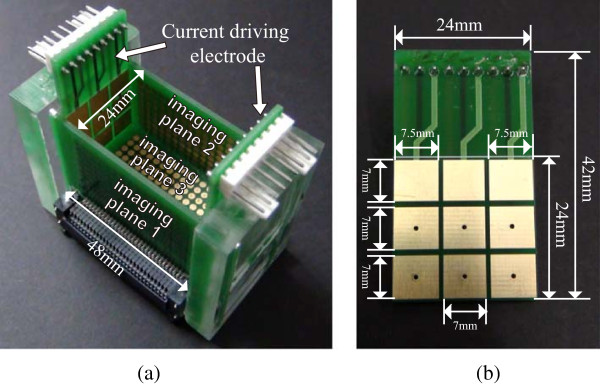
**Sample container and current driving electrodes for producing linearly independent current patterns.** **(a)** The sample container includes the 9 gold coated electrodes used for injecting source and sink currents on the side walls. The 360 voltage sensing electrodes are located on the imaging plane 1, 2, and 3. **(b)** Detailed design and dimensions of the current driving electrodes.

Figure 3 shows the sample container and the design of the current driving electrodes. As explained in the previous section, we needed to control the direction of injection current by the selection of the current driving electrode pairs. This required independent connection of current outputs to current driving electrodes. In the analog backplane, we used low-voltage, T-switches (MAX4529, Maxim, USA) to construct a ‘T’ configuration for handling rail-to-rail signals in either direction. The 360 voltage sensing electrodes on three imaging planes in Figure 1(b) were considered to be a single 24 × 15 electrode array. There are 15 electrodes in each row with a total of 24 rows equally placed in three sides of the container. The first voltmeter measures the differential voltage between electrode 1 and 2 in the same row. From the second voltmeter to the 14th voltmeter, they measure the differential voltage between two adjacent electrodes in a row in the same manner. The 15th voltmeter measures the voltage from electrode channel 15 with reference to the circuit ground. There is an additional measurement circuit used for measuring the amount of applied current inside the current source *via* a current sensing resistor. Each differential recording channel included a variable gain amplifier. The performance characteristics were measured in terms of crosstalk, amplitude stability error (ASE), total harmonic distortion (THD), and signal to noise ratio (SNR) to evaluate the proposed method. The ASE is measured as the standard deviation divided by the mean of the output current amplitude recorded over 1 h. The THD was computed by 5 using 9 harmonic components. The SNR is defined as the ratio of the mean to standard deviation of the repeated measurements.

5

### Phantom experiments

**Figure 4:**
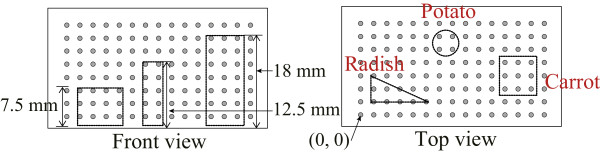
**The phantom design with biological materials.** Three different shapes of biological objects are used as anomalies, pieces of radish, potato, and carrot, to evaluate the performance of measurement method and system.

We filled the container with agar of 5 *Ω*·m resistivity, so that all of the voltage sensing electrodes and current driving electrodes are submerged. The three different current injection methods of , ,  were applied to reconstruct the three projected impedance images on each side. The first current flows between all of the current driving electrodes on the current source plane and current sink plane. This primary current produces a uniform parallel current density distribution inside the container when filled with homogeneous agar. From the results using the primary current, we can calculate the calibration factors for each voltage sensing electrode and measurement channel at different voltmeter gains. After calibrating the system, we placed multiple objects, possessing different shapes and conductivities, into the container. We then obtained the boundary voltage maps from the imaging planes when injecting the primary current. Two secondary currents, used to obtain additional information, are injected between some of current driving electrodes. The direction of each secondary current is independent from the primary current. We measured the boundary voltage maps from the imaging plane 1, 2, and 3 of the container subject to all three different injection currents. Three different biological objects were used as anomalies, pieces of radish, potato, and carrot with the locations shown in Figure 4. The center positions of the radish, potato, and carrot were (5, 5), (14.5, 17), (32, 8.5) mm, respectively. The heights of each object were 7.5, 12.5 and 18 mm as shown in Figure 4. We repeated the measurements subject to each current injection with and without the objects in the container to collect difference data. We measured the resistivity of the background agar (5 *Ω*·m), radish (33 *Ω*·m), potato (40 *Ω*·m), and carrot (50 *Ω*·m) at 1 kHz using an impedance analyzer (1260A, AMETEK Inc. UK) as reference data.

## Results

### Performance evaluation of KHU Mark2 micro-EIT system

**Figure 5:**
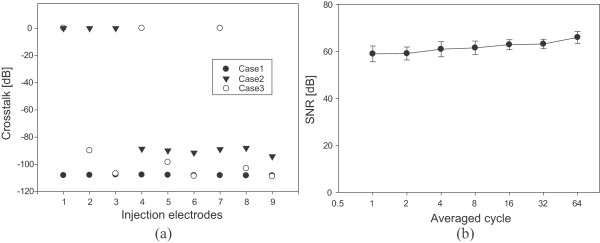
**Performance evaluation of the KHU Mark2 micro-EIT system.** **(a)** The evaluated crosstalk when all switches are in the off-state (case1), when the secondary current flows between  and  (case2), and when the secondary current flows between  and  (case3). **(b)** The averaged SNRs of the 15 measurement channels subject to the number of averaged waveform cycles.

We evaluated the basic performance of the KHU Mark2 micro-EIT system using the container filled with agar. The ASE was 0.016% for 1 h. The THD of the injected current operated at 1 kHz was -110.8 dB. The crosstalk between the adjacent current driving electrodes in all of the current injection cases, is shown in Figure 5(a). Crosstalk between the current driving electrodes was -108 dB when all of the switches for the current injection were in the off-state (case 1). In case 2 and 3, the secondary current was injected between  and  for producing projected images on the imaging plane 1 and 2, and between  and  for one on the imaging plane 3, respectively. The crosstalk of the electrodes positioned around the activated current driving electrodes were -97.2 dB and -90.04 dB for cases 2 and 3, respectively. The crosstalk between the voltmeters and neighboring voltage sensing electrodes was similar to the KHU Mark1 micro-EIT system as reported by Liu *et al.* [23]. The SNR was in the range of 59–66 dB depending on the measurement channels and the number of averaged waveform cycles (Figure 5(b)). The SNR of micro-EIT system was approximately 15 dB lower than the conventional 16 channel EIT system [26, 27]. This degradation was caused by using a large number of small voltage sensing electrodes.

### Phantom experiments

**Figure 6:**
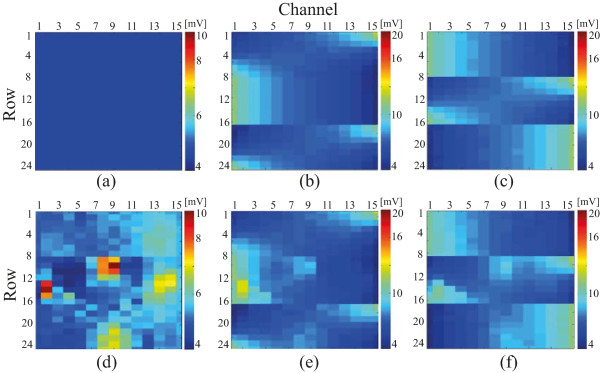
**The measured voltage maps subject to the primary and two different secondary currents.** The voltage distribution on the three imaging planes corresponding to the selection of the current driving electrodes for **(a)** the primary injection, , two secondary injections, **(b)** , and **(c)**  when filling it with homogenous agar. **(d)**–**(f)** are the voltage difference maps of *u*-*u*_0_ when applying primary and two secondary currents with the three objects.

**Figure 7:**
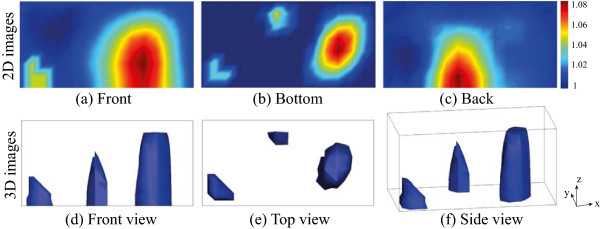
**Projected impedance images on three imaging planes and reconstructed 3D images.** **(a–c)** The two-dimensional projected images of (*γ*_*t*_/*γ*)^-1^ and **(d–f)** the three-dimensional reconstructed images using the projected images of **(a–c)** reconstructed with a back projection algorithm.

Figures 6(a–c) show the measured voltage maps according to the primary, , and two different secondary currents, , , in the homogenous agar container. We could see the current density distribution inside the sample container corresponding to the selection of the current driving electrodes, as expected. Figures 6(d–f) show the voltage difference maps of *u*-*u*_0_ measured by the KHU Mark2 micro-EIT system when applying primary and secondary current in the large-scale container with 3 anomalies. Here, *u* is the measured voltage in the presence of objects seen in Figure 4 and *u*_0_ is the reference voltage without objects. Figures 6, (a) and (d) correspond to the injection current from  to , (b) and (e) correspond to the injection current from  to , and (c) and (f) correspond to the injection current from  to  with and without the anomalies, respectively.The two dimensional impedance images for each of the imaging planes were reconstructed using the projected image reconstruction algorithm in Figures 7(a–c). These were combined with the back projection algorithm to reconstruct three dimensional images. Figures 7(d–f) present three dimensional images in various view angles. The shapes of the three different objects were deformed and blurred from the original shapes because of the intrinsic property of the electric current flow. However, the shapes in the bottom image were consistent with actual shape of the objects in Figure 4. The shape deformation along the z-direction was greater since we did not measure data on the top surface. The center of imaging domain had low sensitivity compared to the boundary region.

### Design of microscopic EIT system for monitoring of tissue culture

**Figure 8:**
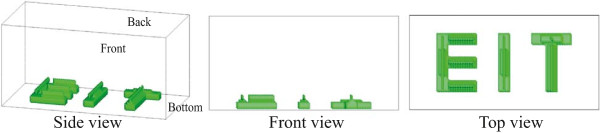
**The simulated model with testing objects in the shape of the letters “EIT”.** The size of the sample container is scaled down by 10 times from the experimental phantom container used earlier. A material of 1.5 *Ω*·m resistivity appears on the bottom side as the shape of “EIT” in a homogeneous material of 1 *Ω*·m resistivity. This material is stacked in a triangular shape from the bottom with a height of 300 *μ*m to simulate the pore space in bone tissue engineering scaffolds where neotissue is grown.

Following the phantom experiment, we undertook a numerical simulation using the proposed electrode configuration with an increasing number of electrodes to determine how many voltage sensing electrodes would be required to perform imaging for the tissue culture. We assumed that the container shown in Figure 1(a) was filled with a homogeneous material of 1 *Ω*·m resistivity. The size of the sample container was scaled down by 10 times. A material of 1.5 *Ω*·m resistivity appeared on the bottom side in the shape of the letters “EIT”. This material was stacked in a triangular shape from the bottom with a height of 300 *μ*m, as shown in Figure 8. The sizes were based on the scaffolds used for bone tissue engineering that typically have a 100–300 *μ*m pore space where neotissue is grown [28].

**Figure 9:**
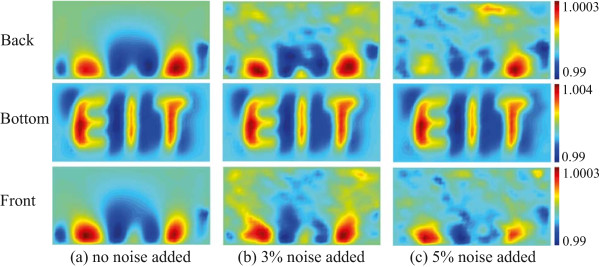
**The reconstructed projection images using 800 voltage electrodes on each side, 2400 in total.** **(a)** no noise added, **(b)** 3% Gaussian random noise added, and **(c)** 5% Gaussian random noise added to the simulated data.

First, we computed the boundary voltage data using equation 1 and the same setup as the experimental system with 360 sensing electrodes (8×15 or 120 sensing electrodes on each side) from the three different current injections. The spatial resolution of the reconstructed impedance image was not enough to recognize the letters because of the distance between the adjacent electrodes and the size of the testing material. The center-to-center spacing of adjacent electrodes was 254 *μ*m and the line width of the letters was 240 *μ*m. For example there was more voxels crossing the edge of ‘E’-letter than voxels wholly within or outside the ‘E’-letter. Therefore, the measured voltage maps from 360 sensing electrodes produced smeared reconstructed images. When we increased the number of voltage sensing electrodes to 20×40 or 800 on each side (2400 in total), the reconstructed projection images show an improved resolution in Figure 9. In this case, the distance between sensing electrodes was 120 *μ*m so that the edges of the letters were defined more clearly. From this observation one can get better resolution when implementing the large number of voltage sensing electrodes. However, the number of voltage sensing electrodes will be limited by the sensitivity of the voltage measurement system and the amount of noise present as the voltage gradient and hence amplitude of measured voltage reduces as the electrodes distance decreases. Further the impedance of the voltage sensing electrodes will increase as they become smaller, placing more challenging requirements on the instrumentation amplifier input impedance for the voltage measurement system.

Based on the results of the system performance test, phantom imaging experiment, and numerical simulations, we can estimate how much the performance will be degraded when miniaturizing the sample container and using more voltage sensing electrodes. Reducing the total volume of the sample container may deteriorate the SNR by 35 dB approximately since the measured signal is decreased by 10 times and the crosstalk is increased due to the high density electrodes for measurements. For this reason, we simulated 3% Gaussian random noise. This is based on an estimate of 30 dB system SNR when miniaturizing the sample container and using the more voltage sensing electrodes. We also considered the increased noise caused by using the smaller size of the sensing electrode. We assumed that this diminished the SNR by 3 dB. Considering both reasons, the case for 5% Gaussian random noise was simulated repeatedly.When the number of electrodes was increased to 2400, Figure 9(a), (b) and (c), the effect of noise on SNR degradation due to the reducing volume and increasing noise is clearly apparent and these considerations need to be made when making a new system to address the larger number of electrodes and smaller container size.

## Discussions

One of the key features in the KHU Mark2 micro-EIT system is the flexible configuration of current driving electrodes that allows the generation of independent current patterns away from sensitive voltage sensing electrodes. For comparison our previous (KHU Mark1) system was limited as the current electrodes were placed in the middle of the array of voltage sensing electrodes [23]. The new electrode configurations and the projected image reconstruction algorithm using a logarithm formulation, instead of using the inverse solver with the least square approximation, may help to avoid a less stable solution and improve the sensitivity of the reconstructed images.

The sensitivity of each voxel is a performance index of how much boundary voltages change due to the admittivity perturbation in that voxel. Therefore, if the sensitivity is too high in one area, there will be a reduced relative sensitivity in other areas. Sensitivity analysis of the KHU Mark2 micro-EIT system showed a relatively uniformly distributed sensitivity in the overall domain and uniform detectability since the current density was more uniform than the Mark1 system. This was an improvement over the previous KHU Mark 1 system which showed an average sensitivity 1.3 times smaller and standard deviation of sensitivity 2 times larger over the imaging domain.We tested the new method in a physical phantom using multiple biological objects such as a radish, carrot, and potato of different sizes in different positions. The proposed method found the exact x-, y-position of tested objects and relative sizes when comparing the tested objects seen in Figure 4 to the reconstructed objects seen in Figure 7. Due to the blurring effect on the reconstructed images the exact shape could not be determined from the images. In order to enhance the quality of reconstructed images along the z-direction, we are considering using the Green functions to reduce the effect of lack of measurements from the top plane.The numerical simulations showed that the proposed method found the shape of objects reasonably well when more sensing electrodes were added and the objects were located closer to the imaging planes. From the numerical simulation results shown in Figure 9, we can expect to continuously monitor regenerated tissue growth inside a scaffold. We may obtain better resolution by implementing a larger number of voltage sensing electrodes. However, the number of voltage sensing electrodes is limited by the noise of the current source, increased noise from smaller electrodes and the voltage measurement noise and sensitivity, as the amplitude of the measurement voltage depends on the distance between adjacent electrodes.

To monitor the cultured tissue in tissue engineering applications, the monitoring system will need to satisfy the following requirements, among others. 1. Sensors or electrodes are embedded in the cultured dish and are operational inside a bioreactor. 2. Since the regenerated tissue will be ultimately implanted to the *in vivo* subject, there is no hazardous effect of electrodes to the growing cells. 3. Commonly, chondrocytes are cultured in a 3D scaffold to form the required, specific structure. 4. Monitoring of this process will require 3D image reconstruction. 5. Large impedance changes will be required, one process that offers this is chondrogenesis, where the cell growth leads to large impedance changes. With these requirements and others satisfied, a system based on the KHU Mark2 micro-EIT system has the potential capability to monitor tissue regeneration within the culture period.

The container implemented in this paper was a macroscopic model for evaluating the performance of the measurement system and confirming the algorithm in the new configuration. We plan to reduce the volume of the sample container using conventional MEMS technology or via a modified MEA as suggested in the numerical simulations. We need further examination and experiments using micro-electrodes with small electrode spacing. Smaller electrodes will have reduced contact area and may cause technical problems related to noise due to current flow or high contact impedance that would deteriorate the performance of the current source and measurement system. A high count data acquisition method with a simple structure is required for accurate and efficient data collection in order to increase the number of voltage sensing electrodes [29].

## Conclusions

We proposed a new micro-EIT system configuration with three linearly independent current patterns and reconstructed projected impedance images without disturbance from secondary current driving electrodes. This avoids some difficulties with previous systems such as an instability of solutions caused by the least-squares method, extra computational cost, and error due to the equipotential plane inside the measurement domain. Overall this improved the quality of the reconstructed images. We evaluated the performance of the KHU Mark2 micro-EIT system using performance metrics: crosstalk, ASE, THD, and SNR. Other performance indexes such as the current source output impedance and CMRR in the voltmeter were similar values reported by Liu *et al.* [22]. Crosstalk between the current driving electrodes existed in the range of -108 to -90.04 dB. Crosstalk produced an error in the current flow and direction, thereby reducing the independence of the primary injection and the secondary injection. With reference to the improved performance and simulation results, we suggested the design and considerations of a micro-EIT system for monitoring of *in vitro* tissue culture. When we considered a large number of voltage sensing electrodes, we could achieve the spatial resolution and sensitivity to monitor the regeneration of articular cartilage.

## Abbreviations

ACI: Autologous chondrocytes implantation
ASE: Amplitude stability error
CMRR: Common mode rejection ratio
EIT: Electrical impedance tomography
KHU: Kyung Hee University
h: Hour
MEA: Multielectrode array
MEMS: Microelectromechanical systems
Micro: Microscopic
SNR: Signal to noise ratio
THD: Total harmonic distortion.
